# One-Step Pharmaceutical Preparation of PEG-Modified Exosomes Encapsulating Anti-Cancer Drugs by a High-Pressure Homogenization Technique

**DOI:** 10.3390/ph16010108

**Published:** 2023-01-11

**Authors:** Tatsuya Fukuta, Mayumi Ikeda-Imafuku, Satoshi Kodama, Junko Kuse, Ko Matsui, Yasunori Iwao

**Affiliations:** 1 Department of Physical Pharmaceutics, School of Pharmaceutical Sciences, Wakayama Medical University, 25-1 Shichiban-Cho, Wakayama 640-8156, Japan; 2 Powrex Corporation, 5-5-5 Kita-Gawara, Itami 664-0837, Hyogo, Japan

**Keywords:** extracellular vesicles, exosomes, high-pressure homogenization, microfluidizer, DDS, anti-cancer drugs, polyethylene glycol, cancer therapy

## Abstract

The use of exosomes encapsulating therapeutic agents for the treatment of diseases is of increasing interest. However, some concerns such as limited efficiency and scalability of conventional drug encapsulation methods to exosomes have still remained; thus, a new approach that enables encapsulation of therapeutic agents with superior efficiency and scalability is required. Herein, we used RAW264 macrophage cell-derived exosomes (RAW-Exos) and demonstrated that high-pressure homogenization (HPH) using a microfluidizer decreased their particle size without changing their morphology, the amount of exosomal marker proteins, and cellular uptake efficiency into RAW264 and colon-26 cancer cells. Moreover, HPH allowed for modification of polyethylene glycol (PEG)-conjugated lipids onto RAW-Exos, as well as encapsulation of the anti-cancer agent doxorubicin. Importantly, the doxorubicin encapsulation efficiency became higher upon increasing the process pressure and simultaneous HPH with PEG-lipids. Moreover, treatment with PEG-modified RAW-Exos encapsulating doxorubicin significantly suppressed tumor growth in colon-26-bearing mice. Taken together, these results suggest that HPH using a microfluidizer could be useful to prepare PEG-modified Exos encapsulating anti-cancer drugs via a one-step pharmaceutical process, and that the prepared functional Exos could be applied for the treatment of cancer in vivo.

## 1. Introduction

Exosomes (Exos) are extracellular vesicles composed of lipid bilayer membranes with particle sizes ranging from 40 to 150 nm and are secreted from almost all types of cells [1]. Exos contain various bioactive components, such as proteins and nucleic acids (including microRNA and mRNA) and are involved in intercellular communication through their transfer to recipient cells [2]. Certain membrane proteins including integrins are known to be expressed on the surfaces of exosomal membranes and have been reported to exert targeting properties derived from parental cells as well as the ability to overcome biological barriers for drug delivery [3,4]. For example, macrophage-derived Exos were reported to target inflamed tumor tissue, similar to homing of macrophages to the tumor tissues [5]. In addition, breast cancer cell-derived and neutrophil-derived Exos were demonstrated to pass through the blood–brain barrier (BBB) [6,7], with the latter also employed to deliver anti-cancer drugs for glioma therapy [7]. Based on the above properties, the application of Exos as natural functional nanoparticles is expected to yield new therapeutics and drug delivery systems (DDS).

Regarding the application of Exos as DDS, encapsulation of both small molecular agents as well as macromolecules (e.g., protein drugs and nucleic acids) has been demonstrated in numerous studies [8,9]. Preparation is typically carried out by incubating a mixture of Exos and drug solution for a certain period of time, especially for hydrophobic small molecular drugs [10,11]. Exosomal membrane permeabilization using a detergent saponin was also combined to increase the encapsulation efficiency of therapeutic agents [12]. Some methods using physical treatments, such as sonication, electroporation, and repeated freeze/thawing have also been reported as a means of loading therapeutic cargos, including proteins and nucleic acid medicines, into Exos [13,14,15]. Other approaches, including co-incubation of donor cells with therapeutic agents to be encapsulated and gene transfection method, have also been reported to harvest drug-encapsulated Exos via purification processes [16,17]. Through these drug encapsulation approaches, the utility of Exos in drug delivery applications has been demonstrated for the treatment of various diseases, including cancers and cardiovascular diseases [15,18]. However, several issues have been pointed out with the above-mentioned methods, such as low drug encapsulation efficiency, poor reproducibility and time-consuming processes, low scalability, risk of Exos aggregation, and influence of certain processes on exosomal properties [8,19]. Therefore, with the ultimate aim of using Exos encapsulating therapeutic agents for clinical applications, new methods considering industrial scale-up, which enables efficient drug encapsulation without adverse influences on exosomal properties, are needed.

To address the above issues, the use of high-pressure homogenization (HPH) with a microfluidizer (Microfluidics, Westwood, MA, USA) has recently been reported as an alternative approach to improve drug encapsulation into Exos with scale-up potential [20]. To date, the utility of HPH has been demonstrated for the production of drug-encapsulated lipid nanoparticles (LNPs), such as liposomes and solid LNPs, due to short production time, lack of a need for organic solvents, and ease of scale-up from lab-scale studies [21,22,23]. Because of these advantages, HPH with microfluidizers is usually employed in the cosmetic and pharmaceutical industries [21]. Our previous studies also applied HPH for the preparation of disk-shaped stable LNPs, called bicelles, and achieved efficient encapsulation of poorly water-soluble drugs [24,25], indicating the further utility of HPH with microfluidizers for preparing nanoparticles composed of phospholipids. Romano et al. found that HPH resulted in efficient encapsulation of the anti-cancer drug irinotecan into glioma cell (U87-MG)-derived Exos compared with incubation alone at 37 °C for 2 h [20]. Moreover, Exos encapsulating irinotecan showed significantly higher cytotoxic effects against U87-MG cells than free irinotecan due to the high cellular uptake ability of Exos in vitro. However, the in vivo efficacy of Exos encapsulating anti-cancer drugs prepared by HPH has not been demonstrated. Moreover, it is still unclear whether the HPH method can be applied to anti-cancer drugs other than irinotecan, as well as whether HPH influences exosomal functionalities (e.g., exosomal marker protein expression and cellular uptake efficiency). Hence, elucidation of these outstanding questions is crucial to demonstrating the further utility of the use of HPH with a microfluidizer for the preparation of Exos encapsulating therapeutic agents.

As one of the issues for in vivo application of Exos, their low blood circulation and targeting abilities due to being trapped by nonspecific organs, especially by the liver and the lung, were pointed out [26,27], despite previous reports suggesting that Exos exhibit targeting properties derived from their parental cells [3,4,5,6,7]. Takahashi et al. previously reported that the negatively charged phospholipid phosphatidylserine, which is known to present abundantly in exosomal membranes, plays a key role in the recognition of Exos by macrophages, resulting in rapid clearance and accumulation in the liver [28,29]. To avoid recognition by macrophages and increase targetability to certain disease sites, surface modification with polyethylene glycol (PEG) is a common strategy in nanoparticle-mediated drug delivery, and the utility of PEG-modified nanoparticle drugs has been demonstrated for the treatment of various diseases [30,31]. It was previously reported that modification of exosomal surfaces with PEG-conjugated phospholipids increased tumor accumulation of intravenously injected Exos via the enhanced permeability and retention (EPR) effects, whereas non-modified Exos was minimally accumulated [32]. Based on these findings, PEG modification of Exos should be useful for the targeted delivery of anti-cancer drugs to tumor tissues, although there are no reports on PEG modification of Exos by HPH. Hence, it is expected that the establishment of a technology that simultaneously allows for PEG modification and drug encapsulation via a one-step pharmaceutical process using HPH could be a powerful avenue for the application of Exos as DDS.

In the present study, we used Exos derived from a murine macrophage-like cell line to investigate the influence of HPH with a microfluidizer on physicochemical properties, expression of exosomal marker proteins, and cellular uptake efficiency. Modification of Exos with PEG and encapsulation of the anti-cancer drug doxorubicin (DOX) by HPH were also investigated. Finally, the in vivo anti-cancer effect of PEG-modified Exos encapsulating DOX was evaluated in tumor-bearing mice. Results of the present study demonstrate the utility of HPH using a microfluidizer for the preparation of functional exosomal formulations by a one-step pharmaceutical process.

## 2. Results

### 2.1. Influence of High-Pressure Homogenization Using a Microfluidizer on Physicochemical Properties of Exosomes

**Figure 1:**
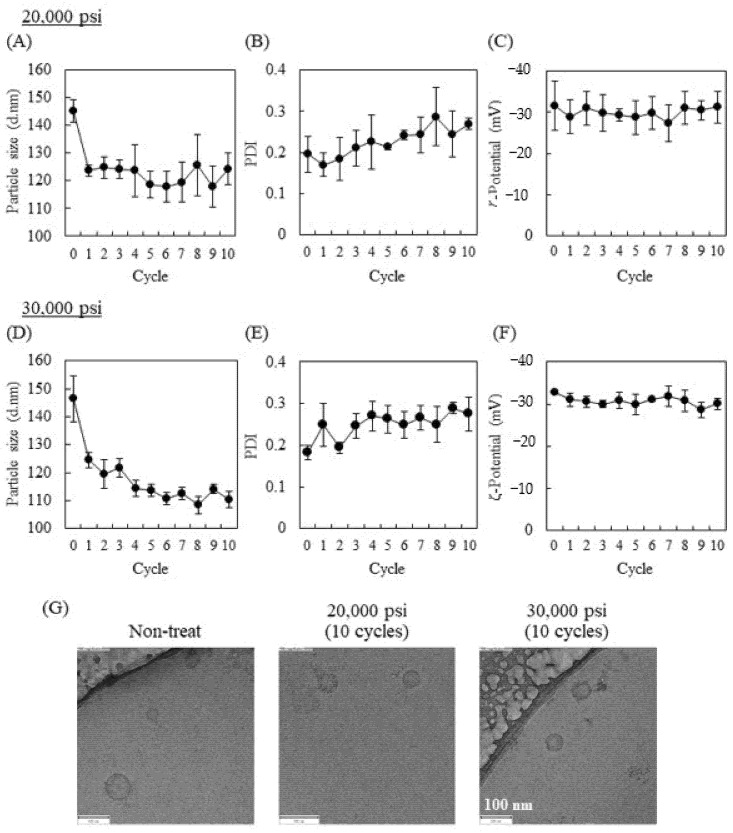
Physicochemical properties of RAW264 cell-derived Exos (RAW-Exos) after each cycle of high-pressure homogenization (HPH) with an LV-1 microfluidizer. (**A**–**F**) Particle size, polydispersity index (PDI), and ζ-potential of RAW-Exos treated by each cycle of HPH at 20,000 (**A**–**C**) or 30,000 psi (**D**–**F**). Data are mean ± S.D. (*n* = 3). (**G**) Cryo-TEM images of non-treated RAW-Exos and RAW-Exos treated by 10 cycles of HPH at 20,000 or 30,000 psi. Scale bars = 100 nm.

Murine macrophage-like RAW264 cell-derived Exos (RAW-Exos) were collected by ultracentrifugation method and used in the following experiments. The average particle size, polydispersity index (PDI), and ζ-potential of the collected RAW-Exos used in the present study were 150.1 ± 4.8 nm, 0.25 ± 0.03, and −30.0 ± 2.8 mV, respectively. The influence of HPH using a microfluidizer on the physicochemical properties of RAW-Exos was first investigated by setting the process pressure at either 20,000 or 30,000 psi. As HPH-mediated irinotecan encapsulation into U87-MG cells (a human glioblastoma cell line)-derived Exos was found to be most efficient at a pressure of 1500 bar (21,750 psi) compared with 500 and 1000 bar in a previous study [20], we used 20,000 psi, a close pressure to 1500 bar, and higher 30,000 psi, a maximum process pressure for the LV-1 microfluidizer. HPH followed by cooling on ice for 5 min is considered 1 cycle. Cooling the samples was €ntended to avoid the denaturation of exosomal components by HPH-induced heat. Using HPH at 20,000 psi, the particle size of RAW-Exos changed from 145.2 ± 4.1 nm to 123.6 ± 2.0 nm after 1 cycle and maintained similar sizes (124.3 ± 5.8 nm at 10 cycles) up to 10 cycles (Figure 1A). PDI tended to increase over 10 cycles of HPH from 0.182 ± 0.018 (Cycle 0) to 0.275 ± 0.04, but was kept <0.3, indicating monodisperse populations (Figure 1B) [33]. The ζ-potential of RAW-Exos at each cycle hardly changed and was maintained at approximately −30 mV, regardless of HPH (Figure 1C). At 30,000 psi, the particle size of RAW-Exos gradually decreased to 110.2 ± 2.9 nm after 10 HPH cycles (Figure 1D). PDI and ζ-potential showed similar tendencies to those of RAW-Exos-treated HPH at 20,000 psi (Figure 1E,F).

As the particle size of RAW-Exos decreased by HPH, we next investigated whether the morphology of RAW-Exos was changed by HPH. To this end, we employed cryo-transmission electron microscopy (TEM), which allows us to directly obtain images of the particles without drying or negative staining [34]. Spherical nanoparticles of approximately 100 nm or less in size were observed in all samples regardless of the use of HPH, which were suggested to be exosomes with sizes of 40–150 nm based on previous reports [1]. Importantly, cryo-TEM images showed almost no apparent differences in the morphological structures of RAW-Exos without HPH compared with those treated with HPH at pressures of 20,000 psi or 30,000 psi (Figure 1G). These results suggest that HPH allows for a reduction in particle size of RAW-Exos without influencing their morphologies.

### 2.2. Effects of HPH on Expression of Representative Exosomal Marker Protein and Cellular Uptake of RAW-Exos

**Figure 2:**
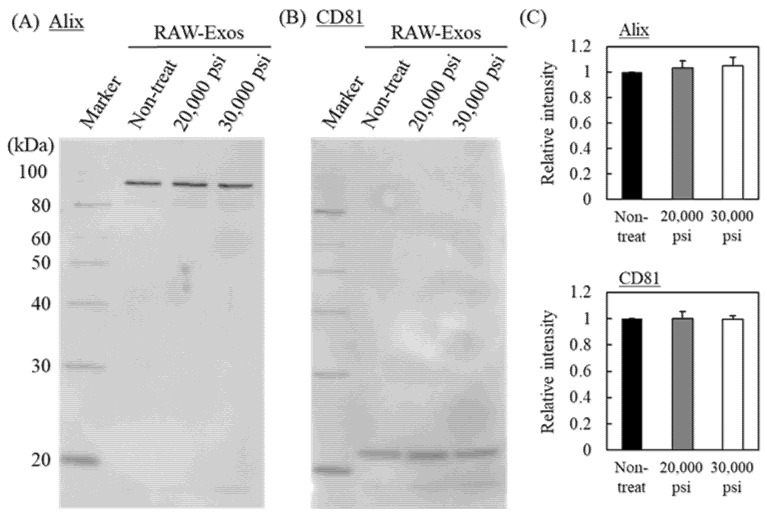
Influence of HPH on exosomal marker proteins. (**A**,**B**) RAW-Exos treated by 10 cycles of HPH at 20,000 or 30,000 psi and non-treated RAW-Exos were subjected to SDS-PAGE (1 µg/lane), followed by Western blotting to observe expression of exosomal markers Alix (**A**) and CD81 (**B**). (**C**) The relative intensities of each band to that of non-treated RAW-Exos are shown for Alix and CD81. Data are mean ± S.D. (*n* = 3).

The influence of HPH on the quality of the RAW-Exos was assessed by evaluating the expression of representative exosomal marker proteins. Although 10 cycles of HPH hardly affected the morphologies of RAW-Exos, as shown in Figure 1, we selected RAW-Exos treated with 10 cycles of HPH to evaluate exosomal marker protein expression. As the marker proteins, Alix and CD81 were selected as proteins present in the internal phase of Exos and that in the lipid membrane, respectively [35]. Results of Western blotting showed similar expression levels of both marker proteins (Alix: 95 kDa and CD81: 22–26 kDa according to the datasheet of the antibody) in RAW-Exos (Figure 2A,B). Relative band intensities of HPH-treated RAW-Exos to non-treated Exos revealed that the amounts of marker proteins remained nearly unchanged after 10 cycles of HPH at 20,000 and 30,000 psi (Figure 2C), suggesting that HPH using a microfluidizer does not induce loss of endogenous proteins entrapped in RAW-Exos.

**Figure 3:**
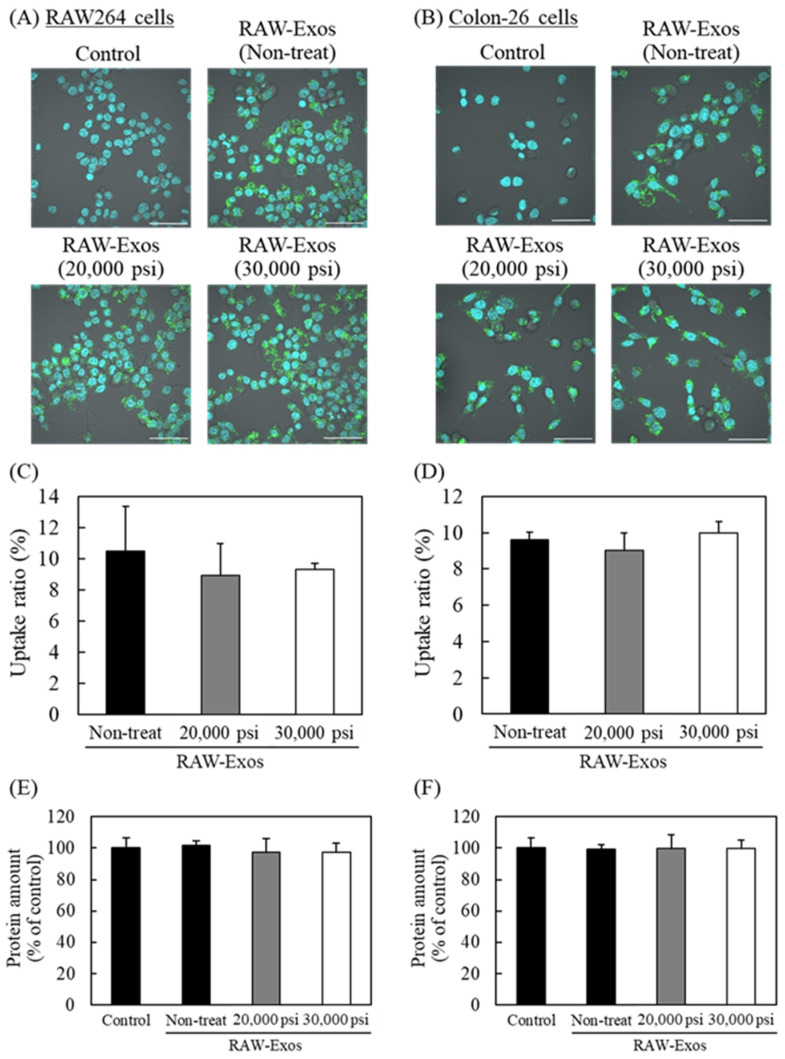
Influence of HPH on cellular uptake of RAW-Exos into parental RAW264 cells and the colon-26 cancer cell line. (**A**,**B**) RAW264 and colon-26 cells were incubated with PKH67-labeled RAW-Exos treated by HPH at each process pressure (5 µg protein/mL). After 6 h incubation, cellular uptake of RAW-Exos was observed by confocal laser scanning microscopy after counterstaining the nuclei with DAPI ((**A**): RAW264, (**B**): colon-26). Green and blue colors indicate fluorescence of PKH67 (RAW-Exos) and DAPI (nuclei), respectively. Scale bars = 50 µm. (**C**,**D**) To quantify the uptake of each sample, the fluorescence intensity of PKH67-labeled RAW-Exos in the cells was measured 24 h after their addition ((**C**): RAW264, (**D**): colon-26). (**E**,**F**) The amount of protein in the cells ((**E**): RAW264, (**F**): colon-26) was determined by the BCA protein assay. Data are mean ± S.D. (*n* = 4).

Next, we assessed the cellular uptake of RAW-Exos to evaluate whether the functionality of the membrane proteins with regard to intracellular uptake could be preserved after HPH treatment, as well as the amount of the exosomal marker proteins. We used parental RAW264 cells and the colon-26 cancer cell line, and RAW-Exos were fluorescently labeled with the fluorescent probe PKH67. Confocal images showed that non-treated RAW-Exos were broadly taken up by their parent RAW264 cells (Figure 3A). Moreover, broad cellular uptake of RAW-Exos treated by HPH at 20,000 or 30,000 psi was observed, similar to that of non-treated RAW-Exos (Figure 3A). The amount of cellular uptake was quantitatively evaluated by lysing the cells; results indicated that nearly similar amounts of RAW-Exos were taken up 24 h after their addition, regardless of HPH (Figure 3C). Similar tendencies were observed for RAW-Exos added to colon-26 cells (Figure 3B,D), suggesting that the membrane protein function of RAW-Exos was not affected by HPH. Finally, treatment with non-treated RAW-Exos and HPH-treated Exos had no effects on the cellular growth of Raw264 and colon-26 cells (Figure 3E,F, respectively).

### 2.3. PEG Modification onto RAW-Exos by HPH

As PEG modification of Exos was previously reported to be important for efficient targeting to tumor tissues [32], we next examined whether HPH enables modification of RAW-Exos with PEG-lipids. As there seemed to be no difference by process pressures in the resultant properties of RAW-Exos, with the exception of particle size, HPH at 30,000 psi was employed for this experiment. To modify RAW-Exos with PEG-lipids, distearoylphosphatidylethanolamine (DSPE)-PEG_2000_ dissolved in PBS was mixed with RAW-Exos at a ratio of 1 µg exosomal protein: 50 µg DSPE-PEG_2000_; this ratio was previously reported to result in better modification efficiency for incubation without HPH, namely incubation for 1 h at 37 °C [32]. HPH-mediated PEG modification was carried out for 10 cycles of HPH at 30,000 psi under coexistence of RAW-Exos and DSPE-PEG_2000_, followed by ultracentrifugation to remove unmodified DSPE-PEG_2000_. As shown in Table 1, PEG modification by 1 h incubation at 37 °C decreased the particle size of RAW-Exos with monodisperse, and their particle size tended to further decrease by HPH, which result corresponded to the results of Figure 1. The ζ-potential of each RAW-Exo maintained a negative charge, while the ζ-potential of PEG-modified RAW-Exos (PEG-RAW-Exos) by HPH became more neutral (Table 1).

**Figure 4:**
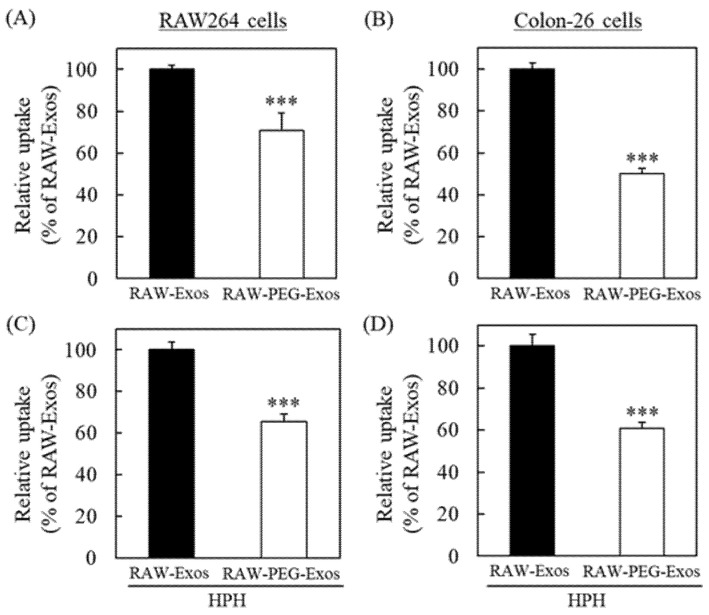
Cellular uptake of PEG-modified RAW-Exos prepared by the HPH method. (**A**,**B**) PKH67-labeled non-treated RAW-Exos or PEG-RAW-Exos prepared by the incubation method were added to RAW264 and colon-26 cells (5 µg protein/mL). (**C**,**D**) PKH67-labeled unmodified-RAW-Exos treated by 10-cycle HPH at 30,000 psi and PEG-RAW-Exos prepared by the HPH method were similarly added to each cell (5 µg protein/mL). At 24 h after incubation, the fluorescence intensity of PKH67 derived from RAW-Exos in the cells was quantified. Data are mean ± S.D. (*n* = 4). *** *p* < 0.001.

By using PEG-RAW-Exos prepared by HPH and the incubation method, we next evaluated their uptake in RAW264 and colon-26 cells and compared them with unmodified RAW-Exos. Each exosomal sample labeled with PKH67 was added to RAW264 or colon-26 cells, followed by lysing after 24 h incubation to quantify the uptake of RAW-Exos. Results indicated that PEG modification onto RAW-Exos by the incubation method significantly decreased uptake into RAW264 and colon-26 cells by 29.1% and 50%, respectively, compared with plain RAW-Exos (Figure 4A,B). Importantly, the uptake of RAW-Exos modified with PEG via the HPH method was also significantly decreased by 34.4% and 39.2% for Raw264 and colon-26 cells (Figure 4C,D), respectively, compared to HPH-treated RAW-Exos without DSPE-PEG_2000_ (Figure 4A,B). We confirmed that PEG modification onto RAW-Exos showed no cytotoxic effect on those cells by the BCA assay. It is known that PEG modification of nanoparticles generally reduces their cellular uptake due to the steric hindrance of the hydrated layers created by PEG [36]. Based on this previous finding, along with the results of the present study, it was demonstrated that HPH using a microfluidizer can be applied to modify RAW-Exos with PEG-conjugated phospholipids.

### 2.4. Encapsulation of the Anti-Cancer Drug Doxorubicin into RAW-Exos by HPH

**Figure 5:**
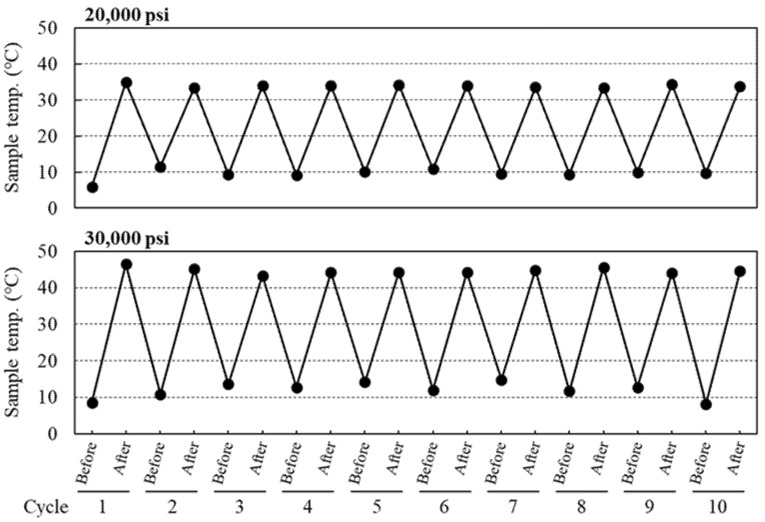
Temperature changes of exosomal samples treated by HPH at each process pressure. The temperature of the exosomal samples was measured just before and after each HPH cycle with process pressures at 20,000 or 30,000 psi.

Next, we investigated whether efficient encapsulation of therapeutic agents can be achieved by HPH. Doxorubicin (DOX) was used as an anti-cancer therapeutic agent, which was previously reported to be encapsulated into Exos by incubation [37] and electroporation [38]. It is well known that temperature is an important process parameter for drug encapsulation into liposomes, and incubation with drugs above the phase transition temperature of the constituent phospholipids leads to an increase in encapsulation efficiency [39]. As Exos are composed of phospholipids [1], similar to liposomes, we hypothesized that transient temperature increases during the HPH cycles would affect drug encapsulation efficiency into Exos. For this purpose, changes in exosomal sample temperature were measured just before and after each HPH cycle at different process pressures. As shown in Figure 5, cycles of 5 min cooling and HPH at each process pressure showed reproducible temperature changes. The average sample temperature changed from 9.6 ± 1.5 °C to 34.0 ± 0.4 °C, and 12.0 ± 2.2 °C to 44.7 ± 0.9 °C at process pressures of 20,000 and 30,000 psi, respectively (Table 2), with the overall change in temperature being higher at the higher process pressure (24.4 ± 1.8 °C and 32.7 ± 2.8 °C at 20,000 and 30,000 psi, respectively (Table 2)).

**Figure 6:**
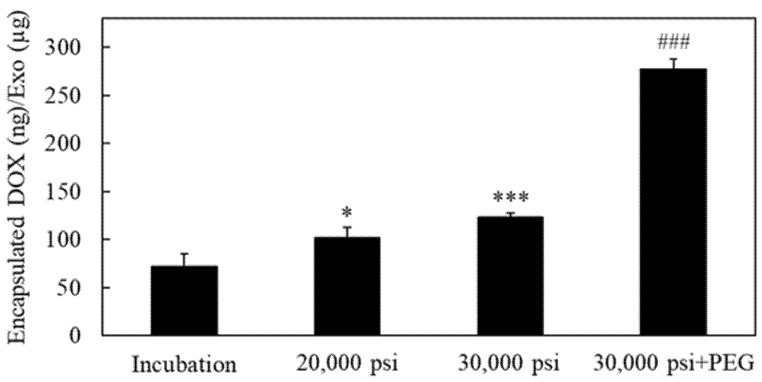
Encapsulation of doxorubicin (DOX) into RAW-Exos by HPH. DOX was encapsulated into RAW-Exos by incubation at 37 °C for 1 h, or 10 cycles of HPH at 20,000 or 30,000 psi. DOX-PEG-RAW-Exos were also prepared by mixing DOX and DSPE-PEG_2000_ into RAW-Exos, followed by 10 cycles of HPH at 30,000 psi. The amount of encapsulated DOX (ng) per amount of initial RAW-Exos (µg protein) is shown. Data are mean ± S.D. (*n* = 3–4). * *p* < 0.05, *** *p* < 0.001 vs. incubation, and ^###^ *p* < 0.001 vs. other groups.

The amount of DOX encapsulated into RAW-Exos by the HPH technique at 20,000 and 30,000 psi was evaluated and compared with that of the incubation method. With incubation alone, the amount of encapsulated DOX was 72.1 ± 12.6 ng per used amount of RAW-Exos (µg protein) (Figure 6). The amount of encapsulated DOX increased significantly to 102.2 ± 10.7 and 123.1 ± 4.1 ng/µg Exos by HPH at 20,000 and 30,000 psi, respectively. Results shown in Figure 5 and Figure 6 indicate that the HPH method increased the encapsulation efficiency of DOX into RAW-Exos compared with incubation alone, and that the degree of temperature change of RAW-Exos might be an important factor affecting the DOX encapsulation efficiency by HPH. Importantly, simultaneous mixing of DSPE-PEG_2000_ with RAW-Exos and DOX solution resulted in significantly higher DOX encapsulation compared with other groups (Figure 6). The DOX encapsulation efficiency (%) of DOX-RAW-Exos prepared by incubation alone, HPH at 20,000, and HPH at 30,000 psi, was 0.43 ± 0.08%, 0.61 ± 0.06%, and 0.73 ± 0.02%, respectively, and that of DOX-PEG-RAW-Exos was 2.85 ± 0.16%. As shown in Table 3, although the average particle size of DOX-RAW-Exos treated by HPH at 20,000 psi or 30,000 psi markedly increased from the results of dynamic light scattering (DLS) measurements, the particle size of PEG-DOX-RAW-Exos (110.8 ± 12.4 nm) was similar to the PEG-RAW-Exos (Table 1). It was previously reported that coating the surface of LNPs with PEG can help avoid aggregation among the particles and increase their stability [40]. Considering this finding, our present results suggest that simultaneous mixing of PEG-lipids into the suspension of RAW-Exos and DOX could suppress aggregation among the RAW-Exos during HPH. On the other hand, it was reported that DSPE-PEG_2000_ and DOX form hydrophobic and electrostatic interactions between the fatty acid chains of DSPE-PEG_2000_ and the anthracycline ring of DOX, and between the phosphate group of DSPE-PEG_2000_ and the amino group of DOX, respectively [41]. These interactions might contribute to an increase in DOX encapsulation into RAW-Exos by simultaneous mixing with DSPE-PEG_2000_.

### 2.5. Anti-Proliferative Effects of DOX-Encapsulated RAW-Exos on Colon-26 Cells

**Figure 7:**
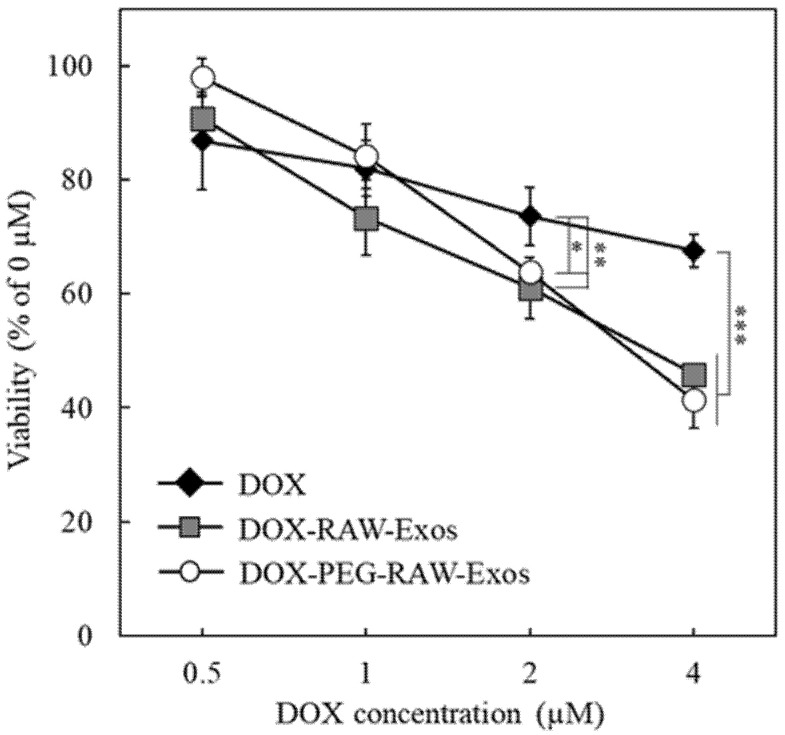
Anti-proliferative effect of DOX-RAW-Exos and PEG-DOX-RAW-Exos on colon-26 cells. colon-26 cells were incubated for 24 h with DOX-PEG-RAW-Exos, DOX-RAW-Exos, or DOX solution at DOX doses of 0.5, 1, 2, or 4 µM. After washing with PBS, the viable cells were determined by a WST-8 assay. Data are mean ± S.D. (*n* = 4). * *p* < 0.05, ** *p* < 0.01, *** *p* < 0.001.

We evaluated the anti-proliferative effects of DOX-RAW-Exos and DOX-PEG-RAW-Exos prepared by HPH at 30,000 psi on colon-26 cells. As HPH at 30,000 psi demonstrated high DOX encapsulation efficiency into RAW-Exos compared with HPH at 20,000 psi (Figure 6), we used DOX-RAW-Exos prepared at 30,000 psi for this experiment. Results showed that DOX-RAW-Exos exhibited significantly higher anti-proliferative effects than DOX solution at DOX concentrations at 2 and 4 µM (Figure 7). DOX-PEG-RAW-Exos also significantly inhibited the growth of colon-26 cells compared with DOX solution at 2 and 4 µM. Since the addition of RAW-Exos onto colon-26 cells had no cytotoxic effect as shown in Figure 3E,F, anti-proliferative effects against the cells are considered to be derived from encapsulated DOX. These results suggest that DOX encapsulated into RAW-Exos was efficiently delivered into colon-26 cells, and that RAW-Exos treated by repeated cycles of HPH could also be applied as useful carriers for intracellular delivery of anti-cancer drugs.

### 2.6. Suppression of Tumor Growth by Treatment with DOX-PEG-RAW-Exos

**Figure 8:**
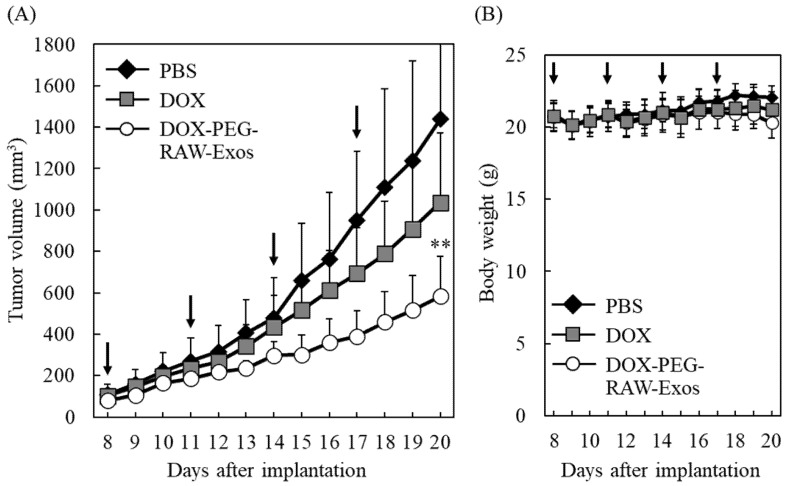
Suppression of tumor growth by DOX-PEG-RAW-Exos in colon-26-bearing mice. (**A**,**B**) BALB/c mice subcutaneously bearing colon-26 cells were intravenously administered DOX-PEG-RAW-Exos (2 mg/kg as DOX dose), DOX solution (2 mg/kg), or PBS at 8, 11, 14, 17 days after tumor implantation. Tumor volume (**A**) and weight of the mice (**B**) were monitored daily. Black arrows in each figure indicate the days of sample injection. Data are mean ± S.D. (*n* = 7–10). ** *p* < 0.01 vs. PBS.

Finally, the antitumor effect of DOX-PEG-RAW-Exos was investigated in colon-26-bearing mice. Due to the average particle size of DOX-RAW Exos being >800 nm, which is beyond the size at which the EPR effect can be exerted [42], along with the previous finding that non-modified plain Exos hardly accumulated in tumor tissue [32], we performed the therapeutic experiment by using DOX-PEG-RAW-Exos. Regarding the DOX dosage, since treatment with PEG-modified liposomal DOX (2 mg/kg as DOX dose) significantly suppressed tumor growth in colon-26-bearing mice in previous reports [43,44], 2 mg/kg DOX dose was considered to be adequate to evaluate the anti-cancer effect of DOX-PEG-RAW-Exos and used in this study. After the tumor volume reached 80–100 mm^3^ (namely, Day 8), treatment with DOX-PEG-RAW-Exos, free DOX, and PBS was carried out four times, namely on days 8, 11, 14, and 17 following tumor implantation. Intravenous administration of four doses of DOX-PEG-RAW-Exos significantly suppressed tumor growth compared with the PBS control group (Figure 8A). Moreover, DOX-PEG-RAW-Exos showed a markedly higher antitumor effect than free DOX. On the other hand, there was almost no difference in body weight between groups (Figure 8B), which was measured as an indicator of systemic side effects.

## 3. Discussion

As Exos can efficiently transfer encapsulated bioactive molecules, such as proteins and nucleic acids, and some species of them also exhibit the ability to overcome biological barriers for drug delivery, their application as endogenous delivery vehicles has attracted considerable attention. However, it was previously noted that conventional drug encapsulation methods suffer from low encapsulation efficiencies, reproducibility, and lack of industrial scale-up methodology for clinical application [8]. Moreover, it is necessary to avoid recognition by macrophages when isolated Exos are injected into the bloodstream, and to increase targetability to desired tissues in vivo [27]. To address these issues, we focused on the use of HPH, which has been successfully used to develop LNP formulations from laboratory scale to industrial scale [21,22,23]. The present study demonstrated that HPH enabled both drug encapsulation and PEG modification into Exos simultaneously via a one-step pharmaceutical process.

In a previous study, HPH at a pressure of 1500 bar (21,750 psi) was used to encapsulate irinotecan into U87-MG-derived Exos, and the cytotoxic effects of the prepared Exos were subsequently demonstrated in vitro, whereas the influence of HPH at a higher process pressure on the physicochemical properties of Exos and on exosomal functionalities was not examined [20]. The present results demonstrate that repeated cycles of HPH at 20,000 psi and 30,000 psi (maximum process pressure) decreased the average particle size of RAW-Exos to approximately 120 and 110 nm, respectively, without apparent influences on the exosomal morphologies (Figure 1). One possible reason for the decrease in average particle size by repeated HPH cycles may be that aggregates formed between populations of smaller RAW-Exos and populations of larger RAW-Exos were loosened by shear forces at the interaction chamber walls in the microfluidizer during the HPH processes [23]. On the other hand, the amounts of exosomal marker proteins Alix and CD81, which were respectively observed as representative proteins presenting in the internal phase and in the lipid membranes of Exos, hardly changed after HPH (Figure 2), suggesting that physical stimulation by HPH could not induce leakage of exosomal contents and membrane damage. In addition, no significant effects were observed by HPH on the uptake of RAW-Exos into parental RAW264 cells and colon-26 cells (Figure 3), suggesting that HPH did not induce denaturation or decreases in the function of exosomal membrane proteins involved in cellular uptake. Collectively, these results indicate that HPH using a microfluidizer resulted in decreasing particle sizes of Exos without changing their inherent properties.

For LNP formulations composed of phospholipids similar to Exos, PEG modification has become the gold standard to improve targetability by avoiding macrophage recognition and increasing formulation stability [30,40]. Results of the present study demonstrate that PEG-lipids could be easily inserted into RAW-Exos during the HPH process, resulting in reduced uptake by macrophages in vitro (Figure 4). In the field of LNP research, PEG-lipids conjugated with reactive groups, such as N-hydroxysuccinimide and maleimide, have been widely used for modification of targeting ligands, which allows for ligand-mediated targeting of the resultant LNP to specific cells and organs [45,46]. Based on the results of this study, it is expected that Exos can also be modified with targeting ligand-conjugated PEG-lipids by HPH, which enables Exos to not only avoid macrophage uptake but also increase the targeting ability to desired sites. To further demonstrate the usefulness of HPH for exosomal engineering, use of HPH to prepare targeting ligand-conjugated Exos could be an interesting focus of future research.

HPH was found to significantly increase the DOX encapsulation efficiency compared with incubation alone, with the encapsulation efficiency being higher at a process pressure of 30,000 psi compared with 20,000 psi (Figure 6). Moreover, sample temperatures were also found to increase more with increasing process pressure, with the highest temperature change being observed after HPH at 30,000 psi (Figure 5 and Table 2). It was previously reported that increasing the fluidity of liposomal lipid membranes by incubation above the phase transition temperature of the constituent phospholipids is a crucial factor for drug encapsulation into liposomes [39]. As there was a difference of 11.3 °C in sample temperature between HPH at 20,000 and 30,000 psi, we speculate that the HPH-induced transient increase in both exosomal temperature and membrane fluidity might be one of the mechanisms that brought about an increase in DOX encapsulation into RAW-Exos. With regard to the lipid composition of Exos, previous reports indicated that exosomal membranes contain a high amount of cholesterol (ca. 40–43%) and are also composed of phospholipids such as sphingomyelin, phosphatidylcholine, and phosphatidylserine, whose percentages depend on the specific cellular species [47,48]. Cholesterol regulates lipid membrane fluidity through interaction with sphingomyelin and phospholipids [49] and decreases membrane permeability by increased packing between the lipids [50]. Nevertheless, HPH using a microfluidizer achieved efficient DOX encapsulation, suggesting that shear forces caused by HPH might transiently disturb the rigid exosomal membranes and contribute to the permeation of DOX molecules into RAW-Exos. In addition, the simultaneous mixing of DSPE-PEG_2000_ significantly increased DOX encapsulation compared with HPH alone, without increasing the average particle size of RAW-Exos (Figure 6 and Table 2). As mentioned in Section 2.4, DSPE-PEG_2000_ and DOX were reported to form hydrophobic and electrostatic interactions at physiological pH between the fatty acid chains of DSPE-PEG_2000_ and the anthracycline ring of DOX, and between the phosphate group of DSPE-PEG_2000_ and the amino group of DOX, respectively [41]. Similar interactions were previously observed in the preparation of PEG-modified liposomes encapsulating nystatin, a drug that also possesses hydrophobic moieties and an amino group [51]. Based on these findings, it is suggested that DOX was incorporated into RAW-Exos while interacting with DSPE-PEG_2000_, resulting in an increase in the DOX encapsulation efficiency by simultaneous HPH with DOX and DSPE-PEG_2000_.

Finally, we showed that treatment with DOX-PEG-RAW-Exos significantly suppressed tumor growth and exerted a stronger anti-cancer effect compared with free DOX in colon-26-bearing mice (Figure 8) Based on the previous finding that PEGylated Exos accumulates in tumor tissue by the EPR effect [32], it is suggested that DOX-PEG-RAW-Exos could also accumulate in tumor tissue via the EPR effect, and that DOX released from the PEG-RAW-Exos could then exert its cytotoxic effect. Moreover, as PEG-RAW-Exos were taken up by colon-26 cells and DOX-PEG-RAW-Exos showed a cytotoxic effect similar to DOX-RAW-Exos in vitro (Figure 4 and Figure 7), the DOX-PEG-RAW-Exos accumulated in the tumor tissue were suggested to be taken up by the cancer cells via membrane proteins presenting on the RAW-Exos and showed a growth inhibition effect in vivo. Taken together, the results of the present study suggest that DOX-PEG-RAW-Exos prepared by HPH should function not only in vitro but also in vivo, and that the exosomal formulations could be useful for cancer treatment.

In conclusion, our present study demonstrated that HPH with a microfluidizer can be used to decrease the particle size of RAW-Exos without affecting their morphology and apparent functionality. Use of HPH was found to efficiently encapsulate the anti-cancer drug DOX into RAW-Exos and allowed for modification with PEG. Moreover, DOX and PEG-lipids could be simultaneously loaded into RAW-Exos via a one-step pharmaceutical process using a microfluidizer, and the resultant DOX-PEG-RAW-Exos significantly suppressed tumor growth in vivo. Based on these findings, we suggest that application of HPH with a microfluidizer to Exos could be useful for the preparation of functional exosomal formulations via a one-step pharmaceutical process. To the best of our knowledge, this is the first report demonstrating the effectiveness of PEG-modified Exos encapsulating anti-cancer drugs prepared by HPH.

## 4. Materials and Methods

### 4.1. Cell Cultures

The murine macrophage-like cell line RAW264 and the murine colon adenocarcinoma cell line colon-26 were purchased from RIKEN Cell Bank (Tsukuba, Japan). RAW264 cells were cultured in Dulbecco’s Modified Eagle’s Medium (DMEM; Nacalai Tesque, Kyoto, Japan) supplemented with 10% fetal bovine serum (FBS), 100 U/mL penicillin and 100 µg/mL streptomycin (Gibco, Waltham, MA, USA). Colon-26 cells were cultured in RPMI1640 (Nacalai Tesque) supplemented with 10% FBS, 100 U/mL penicillin, and 100 µg/mL streptomycin. These cell lines were cultured at 37 °C in a 5% CO_2_ incubator.

### 4.2. Isolation of Exosomes

RAW264 cells were seeded onto 150-mm dishes (9 × 10^6^ cells/dish) to result in 80% confluency after overnight incubation. The medium was then aspirated, and the cells were washed with phosphate-buffered saline (PBS). Culture medium was replaced with serum-free Advanced DMEM (Gibco) supplemented with 2 mM L-glutamine (Gibco), 100 U/mL penicillin, and 100 µg/mL streptomycin. At 48 h after additional incubation, the culture supernatant was harvested, and RAW264-derived exosomes (RAW-Exos) in the medium were isolated as described previously [52]. Briefly, the supernatant was sequentially centrifuged at 300× *g* for 10 min, 2000× *g* for 20 min, and 10,000× *g* for 30 min at 4 °C (Allegra X-30R; Beckman Coulter, Tokyo, Japan), followed by filtration using 0.22-µm syringe filters (Merck Millipore, Billerica, MA, USA). The samples were then ultracentrifuged at 125,000× *g* for 70 min at 4 °C (Optima XE-90; Beckman Coulter) to pellet the RAW-Exos, resuspended in PBS, and subjected to ultracentrifugation (125,000× *g*, 70 min, 4 °C). The resultant RAW-Exos were resuspended in PBS and stored at −80 °C before use in the following experiments.

### 4.3. Measurement of Physicochemical Properties and Protein Concentration of RAW-Exos

The particle size, PDI, and ζ-potential of exosomal samples were determined with a Zetasizer Pro (Malvern Instruments, Malvern, Worcestershire, UK). The protein concentration of the RAW-Exos in suspension was measured using a Micro bicinchoninic acid (BCA) Protein Assay Kit (Pierce Biotechnology, Rockford, IL, USA) in accordance with the manufacturer’s instruction. Absorbance was measured at 562 nm using a microplate reader (Tecan Infinite M Plex, Tecan Japan, Kanagawa, Japan).

### 4.4. High-Pressure Homogenization (HPH) with a Microfluidizer

For treating RAW-Exos by high-pressure homogenization (HPH), 3 mL of 120 µg/mL RAW-Exos suspended in PBS was prepared, and an LV-1 microfluidizer having a Y-type interaction chamber (Microfluidics, Westwood, MA, USA) was employed. Samples were subjected to HPH at 20,000 or 30,000 psi with 0–10 cycles, and 100 µL aliquots were collected for each cycle. Immediately after HPH at each cycle, samples were cooled on ice for 5 min. The physicochemical properties of the aliquots were analyzed with the Zetasizer Pro as described above.

### 4.5. Cryo-Transmission Electron Microscopy

Cryo-TEM images were acquired on a JEM-2100Plus microscope (JEOL Co., Ltd., Tokyo, Japan). A 2 μL aliquot of RAW-Exos and RAW-Exos with HPH suspension was then applied to the hydrophilic 200-mesh copper grid. The grid was then blotted with filter paper for 3 s and immediately vitrified in liquid ethane cooled with liquid nitrogen, using a Leica EM-GP II cryofixation system (Leica Microsystems GmbH, Wetzlar, Germany). Frozen samples were maintained at −170 °C, using an Elsa Cryo-Transfer Holder, model 698 (Gatan, Inc., Pleasanton, CA, USA). The cryo-TEM was operated at 200 kV and provided a magnification of ×80,000.

### 4.6. Western Blotting

To observe the influence of HPH on exosomal marker protein expression, RAW-Exos treated with 10 cycles of HPH or non-treated RAW-Exos were enriched by ultracentrifugation (125,000× *g*, 70 min, 4 °C). Protein concentrations were then measured using a Micro BCA Protein Assay Kit. The resultant RAW-Exos (1 µg protein) were subjected to 10% SDS-PAGE and transferred electrophoretically to a polyvinylidene difluoride (PVDF) membrane (Bio-Rad, Hercules, CA, USA). After blocking for 1 h at 37 °C with 3% bovine serum albumin (BSA) in Tris-HCl-buffered saline containing 0.1% Tween20 (pH 7.4), the PVDF membrane was reacted with anti-Alix mouse antibody (sc-53540; Santa Cruz Biotechnology, Santa Cruz, CA, USA) or anti-CD81 mouse antibody (sc-166029; Santa Cruz Biotechnology) at a dilution of 1:100 at 4 °C overnight, respectively. The membrane was then incubated with HRP-conjugated rabbit anti-mouse IgG (ab97046; Abcam, Cambridge, UK) at a dilution of 1:20,000 for 1 h at 37 °C. After reaction with Amersham ECL Prime Western Blotting Detection Reagent (Tokyo, Japan), protein bands were acquired with an ImageQuant LAS 500 (Cytiva). Band intensities were quantified using the image analysis software ImageJ (National Institutes of Health, Bethesda, MD, USA).

### 4.7. Cellular Uptake of RAW-Exos

For evaluation of cellular uptake of RAW-Exos, RAW-Exos treated with 10 cycles of HPH (20,000 or 30,000 psi) and non-treated RAW-Exos were fluorescently labeled with PKH67 (Sigma-Aldrich, St. Louis, MO, USA) according to the manufacturer’s protocol. PKH67-labeled RAW-Exos were washed twice with PBS using Amicon Ultra 10K (Merck Millipore).

RAW264 cells (5.0 × 10^5^ cells/dish) or colon-26 cells (2.0 × 10^5^ cells/dish) were seeded onto a 35 mm glass bottom dish and incubated at 37 °C overnight. After removal of the medium, PKH67-labeled RAW-Exos were added to the RAW264 or colon-26 cells at a final concentration of 5 µg exosomal protein/mL in 10% FBS containing DMEM or RPMI1640, respectively. After 6 h incubation, cells were washed twice with PBS and fixed with 4% paraformaldehyde for 10 min at 37 °C. Then, the nuclei of the cells were stained with 1 µg/mL 4′,6-diamidino-2-phenylindole (DAPI; Thermo Fisher Scientific, Waltham, MA, USA) in PBS for 15 min at 37 °C, followed by observation of the fluorescence by confocal laser scanning microscopy (FV3000, Olympus, Tokyo, Japan).

For quantitative analyses, RAW264 cells (2.0 × 10^4^ cells/well) or colon-26 cells (2.0 × 10^4^ cells/well) were seeded in a 96-well black plate and incubated at 37 °C overnight. Following treatment with each PKH67-labeled RAW-Exos for 24 h, cells were washed with PBS and lysed with 1% n-octyl-β-D-glucoside (Dojindo, Kumamoto, Japan). The PKH67 fluorescence intensity was measured with a Tecan Infinite M Plex microplate reader. Thereafter, the protein contents of each cell were determined with a BCA protein assay reagent kit (Pierce Biotechnology) as an indicator of cellular viability.

### 4.8. Modification of RAW-Exos with PEG-Lipid

RAW-Exos and distearoylphosphatidylethanolamine (DSPE)-PEG_2000_ (Nippon Fine Chemical, Hyogo, Japan) dissolved in PBS were mixed at a ratio of 1 µg exosomal protein: 50 µg DSPE-PEG_2000_, a previously reported ratio for efficient PEG modification [32]. The mixture of RAW-Exos and DSPE-PEG_2000_ was subjected to 10 cycles of HPH at 30,000 psi and 5 min cooling as described above (Section 4.4), followed by ultracentrifugation (125,000× *g*, 70 min, 4 °C) to remove unmodified DSPE-PEG_2000_. In the case of PEG modification without HPH, the mixture was incubated for 1 h at 37 °C as previously reported [32]. After ultracentrifugation, the pellets were resuspended in PBS, and the physicochemical properties of the resultant PEGylated RAW-Exos (PEG-RAW-Exos) were measured using the Zetasizer Pro. To compare the cellular uptake efficiency of PEG-RAW-Exos prepared by each method, the samples were labeled with PKH67, and RAW264 cells or colon-26 cells seeded in 96-well black plate were treated with PKH67-labeled PEG-RAW-Exos as described in Section 4.7.

### 4.9. Measurement of Sample Temperature after HPH

Temperature changes of exosomal samples following repeated cycles of HPH, namely 10 cycles of HPH at 20,000 or 30,000 psi and subsequent 5 min cooling on ice, were monitored with a Waterproof Digital Thermometer SN-3000 (NETSUKEN, Tokyo, Japan). The temperature of the samples was measured just before and after HPH by directly inserting the probe into a 5 mL lock syringe (TOP, Tokyo, Japan) containing 3 mL of exosomal samples.

### 4.10. Doxorubicin Encapsulation into RAW-Exos by HPH

To encapsulate doxorubicin hydrochloride (DOX; Cayman Chemical, Ann Arbor, MI, USA) into RAW-Exos, exosomal suspensions and DOX dissolved in PBS were mixed to prepare 120 µg RAW-Exos/1 mg DOX/1 mL PBS samples. For preparation of DOX-PEG-RAW-Exos, 217 µL of 10 mM DSPE-PEG_2000_ (6 mg) dissolved in ultrapure water was also mixed in the initial samples to yield a ratio of 1 µg exosomal protein:50 µg DSPE-PEG_2000_, as described above. Then, each sample was subjected to 10 cycles of HPH at 20,000 or 30,000 psi, followed by ultracentrifugation (125,000× *g* for 70 min at 4 °C) to remove unencapsulated DOX and unmodified PEG-lipids. For the Exos prepared by the incubation method, the exosomal samples were incubated at 37 °C for 1 h and purified by ultracentrifugation. The resultant pellets were resuspended in PBS. The amount of the encapsulated DOX was determined by measuring absorbance at 484 nm after solubilizing the exosomal samples in 1% Triton X-100 at 37 °C for 10 min. The DOX encapsulation efficiency was calculated as the percentage of the amount of encapsulated DOX in RAW-Exos to the amount of DOX added to the initial RAW-Exos.

### 4.11. In Vitro Cytotoxicity Assay

Colon-26 cells (2.0 × 10^4^ cells/well) were seeded in a 96-well plate and cultivated overnight. DOX-PEG-RAW-Exos, DOX-RAW-Exos, or DOX solution containing DOX doses of 0.5, 1, 2, and 4 µM in DMEM FBS (+) were added to the cells and incubated for 24 h. After washing with PBS, cell viability was assessed by a WST-8 assay, in which FBS-free DMEM containing 10% Cell Counting Kit-8 (Dojindo) was added to the medium and incubated for 1 h at 37 °C. Thereafter, absorbance of the sample (λ = 450 nm) and reference (λ = 630 nm) was measured by the microplate reader.

### 4.12. In Vivo Anti-Cancer Experiments

BALB/c mice (male, 5 weeks old) were purchased from Japan SLC, Inc. (Shizuoka, Japan). All animal protocols were reviewed and approved by the Animal and Ethics Review Committee of Wakayama Medical University. Mice were housed under 12 h light/12 h dark cycles and provided free access to water and feed. Colon-26 cells (2 × 10^6^ cells/mouse) were subcutaneously implanted into the left posterior flank to prepare subcutaneous tumor-bearing mice as previously described [53]. When tumor volume reached 80–100 mm^3^ (8 days after implantation of tumor cells), treatment with DOX-PEG-RAW-Exos (2 mg/kg as DOX dose), DOX solution (2 mg/kg), or PBS was initiated, and the mice were intravenously administered the treatment sample on days 8, 11, 14, 17 after tumor implantation. Monitoring of tumor volume was performed daily, and tumor volume was calculated according to the following equation: 0.4 × a × b^2^ (a, the largest diameter; b, the smallest diameter). Body weight was also measured daily, as an indicator of common side effects of anti-cancer drugs.

### 4.13. Statistical Analysis

Statistical differences between groups were determined by one-way analysis of variance with the Tukey post hoc test. Differences between the two groups were evaluated using the Student’s *t*-test. Data are presented as the mean ± S.D.

## Figures and Tables

**Table 1:** Physicochemical properties of RAW264 cell-derived exosomes (RAW-Exos) and PEG-RAW-Exos prepared by the conventional incubation method or HPH using a LV-1 microfluidizer (*n* > 4).

Sample	Particle Size (d.nm)	Polydispersity Index (PDI)	ζ-Potential (mV)
RAW-Exos	150.1 ± 4.8	0.25 ± 0.03	−30.0 ± 2.8
PEG-RAW-Exos (Incubation)	125.6 ± 12.2	0.20 ± 0.05	−29.4 ± 5.9
PEG-RAW-Exos (HPH)	113.2 ± 27.6	0.29 ± 0.04	−17.4 ± 1.6

**Table 2:** Average temperature of exosomal samples treated before/after HPH at each process pressure (*n* = 10).

Process Pressure (psi)	Before (°C)	After (°C)	Degree of Temperature Change (°C)
20,000	9.6 ± 1.5	33.4 ± 0.4	24.4 ± 1.8
30,000	12.0 ± 2.2	44.7 ± 0.9	32.7 ± 2.8 ***

*** *p* < 0.001 vs. 20,000 psi.

**Table 3:** Physicochemical properties of doxorubicin (DOX)-encapsulated RAW-Exos prepared by HPH (*n* = 3–5).

Sample	Particle Size (d.nm)	Polydispersity Index (PDI)	ζ-Potential (mV)
DOX-RAW-Exos (20,000 psi)	1222.7 ± 187.0	0.38 ± 0.15	−19.8 ± 7.2
DOX-RAW-Exos (30,000 psi)	887.2 ± 445.6	0.49 ± 0.16	−21.3 ± 3.8
DOX-PEG-RAW-Exos	110.8 ± 12.4	0.35 ± 0.04	−27.5 ± 1.3

## Data Availability

The data presented in this study are available from the corresponding authors upon reasonable request.
